# Presence of exoY, exoS, exoU and exoT genes, antibiotic resistance and biofilm production among Pseudomonas aeruginosa isolates in Northwest Iran

**DOI:** 10.3205/dgkh000264

**Published:** 2016-02-22

**Authors:** Somayeh Azimi, Hossein Samadi Kafil, Hossein Bannazadeh Baghi, Saeed Shokrian, Khadijeh Najaf, Mohammad Asgharzadeh, Mehdi Yousefi, Firooz Shahrivar, Mohammad Aghazadeh

**Affiliations:** 1 Drug Applied Research Center, Tabriz University of Medical Sciences, Tabriz, Iran; 2 Immunology Research Center, Tabriz University of Medical Sciences, Tabriz, Iran; 3 Infectious Disease and Tropical Medicine Research Center, Tabriz University of Medical Sciences, Tabriz, Iran; 4 Biotechnology Research Center, Tabriz University of Medical Sciences, Tabriz, Iran; 5 Department of Medical Microbiology and Virology, Faculty of Medicine, Tabriz University of Medical Sciences, Tabriz, Iran

**Keywords:** Pseudomonas aeruginosa, infection, biofilm, exo genes, type III secretion system

## Abstract

**Background:** *Pseudomonas aeruginosa*, as Gram-negative rod bacilli, has an important role in human infection. In the present study we aimed to investigate the presence of *exo* genes and biofilm production among *Pseudomonas aeruginosa* isolates in Northwest Iran.

**Material and methods:** 160 isolates of *P. aeruginosa* were collected and identified by biochemical tests and were characterized for antibiotic resistance. Biofilm production was evaluated by microtiter plate assay and the presence of *exo* genes was evaluated by allele-specific PCR (polymerase chain reaction). Chi-square test was used for statistical analysis.

**Results:** The most effective antibiotics against isolates were colistin and polymyxin B. 87% of the isolates were biofilm producers of which 69% were strongly biofilm producers. 55% of the isolates carried *exoY*, 52% of the isolates carried *exoU*, and 26.3% and 5% carried *exoS* and *exoT*, respectively.

**Conclusion:** Our findings showed different distribution of *exo* genes in clinical isolates of *P. aeruginosa* in Northwest Iran. *ExoS* and *exoU* were more prevalent in non-biofilm producers and *exoY* was more prevalent in biofilm producer isolates. These results might indicate the importance of *exoY* in biofilm production of *Pseudomonas aeruginosa*.

## Zusammenfassung

**Hintergrund:** *Pseudomonas aeruginosa*, ein Gram-negatives Stäbchenbakterium, besitzt eine wichtige Rolle als Krankheitserreger. Daher untersuchten wir *Pseudomonas aeruginosa*-Isolate im Nordwestiran auf das Vorkommen von *exo*-Genen und Biofilmbildnern.

**Material und Methode:** 160 *P. aeruginosa*-Isolate wurden biochemisch identifiziert und die Antibiotikaresistenz charakterisiert. Die Fähigkeit zur Biofilmbildung wurde im Mikrotiterplatten-Assay, das Vorkommen von *exo*-Genen mit Allel-spezifischer PCR (Polymerase-Kettenreaktion) analysiert. Zur statistischen Analyse wurde der Chi-Quadrat-Test eingesetzt.

**Ergebnisse:** Als effektivste Antibiotika erwiesen sich Colistin und Polymyxin B. 87% der Isolate waren Biofilmbildner, davon 69% mit massiver Biofilmbildung. In 55% der Isolate wurden *exoY*, in 52% *exoU*, in 26,3% *exoS* und in 5% *exoT* nachgewiesen.

**Schlussfolgerung:** Die Analyse ergab eine unterschiedliche Verteilung der *exo*-Gene bei klinischen Isolaten von *P. aeruginosa* im Nordwestiran. *ExoS* und *exoU* kamen häufiger bei nicht biofilmbildenen Isolaten, *exoY* häufiger bei Biofilmbildnern vor. Die Ergebnisse können ein Hinweis auf die Bedeutung von *exoY* bei Biofilm bildenden *Pseudomonas aeruginosa*-Isolaten sein.

## Introduction

*Pseudomonas aeruginosa* is an important causative agent of human infection, especially in a host with compromised defense mechanisms [1]. This bacterium has minimal nutritional requirements, tolerates a wide variety of physical conditions [2], [3] and forms biofilm on the biotic or abiotic surface [4]. In human hospitals, *Pseudomonas* is a leading cause of nosocomial infections via colonization of catheters, skin wounds, ventilator-associated pneumonia, and is also a cause of respiratory infections in individuals with cystic fibrosis (CF) [5]. Colonization by *Pseudomonas* spp. occurs when the fibronectin coat surrounding host cells is destroyed due to trauma or infection [6].

The virulence factors can be chemical or proteinaceous, and either cell-associated or secreted. Proteinaceous virulence factors are often secreted through one of the five protein secretion systems so far described as *P. aeruginosa*: type I, II, III, V [7] and the recently discovered type VI [8]. Especially the type III secretion system (TTSS), which injects effector proteins directly into the eukaryotic host cell cytoplasm, has been associated with high virulence. Infection with a type III secreting isolate has been shown to correlate with severe disease [9], and type III secretion (TTS) in lower respiratory and systemic infections is associated with an increased mortality rate.

*P. aeruginosa* has an impressive array of cell-associated and secreted virulence factors that contribute to its pathogenesis. Key among these is type IV pili, the major bacterial adhesion factor, and the type III secretion system with its secreted exotoxins [10]. Upon host cell contact, the type III secretion system allows bacteria to directly inject toxins into the host cell, where they subvert host cell defense and signaling systems [11]. Four type III-secreted effectors have been identified in *P. aeruginosa*, although few if any strains secrete all four of them [12]. 

*ExoU* is a potent cytotoxin whose host cell targets and mechanism of action are not yet known [13]. *ExoT* is a bifunctional protein, possessing an N-terminal GTPase-activating domain with GAP (G-protein-activating protein) activity toward Rho, Rac, and Cdc42, and a C-terminal ADP-ribosyltransferase domain [14].

*ExoS* and *exoT* are highly homologous bifunctional proteins with an amino terminal GAP domain and a carboxy-terminal ADP-ribosylation domain [15], [16]. In the present study we aimed to investigate the presence of *exo* genes and biofilm production among *Pseudomonas aeruginosa* isolates in Northwest Iran.

## Methods and materials

### Bacterial isolates and identification of Pseudomonas aeruginosa

A total of 160 *P. aeruginosa* isolates were collected from wounds, respiratory tract, urinary tract, blood stream and sputum of patients admitted to Imam Reza, Shaheed Madani, and Sina hospitals in Tabriz during September 2013 to July 2014. The isolates were confirmed as *P. aeruginosa* by colony morphology, motility, pigment production, growth at 42°C and 4°C, Gram staining, and conventional biochemical tests [17].

### Antibiotic susceptibility tests 

Antimicrobial susceptibility of the isolates against 11 antibiotics was performed by the Kirby-Bauer disk diffusion method on Muller-Hinton agar in order to determine the resistance pattern according to the CLSI (Clinical and Laboratory Standards Institute) guideline [18]. The susceptibility and resistance of *P. aeruginosa* to the following antibiotic disks were tested: amikacin, cefepime, ceftazidime, tobramycin, gentamicin, imipenem, colistin, ciprofloxacin, piperacillin, gatifloxacin and polymyxin B (antibiotic selection was according to CLSI recommendation and local use of antibiotic in this region). The interpretation of sensitivity was done according to the CLSI breakpoint. *P. aeruginosa* (ATCC 27853) was used for quality control.

### Protocol preparation of bacterial DNA

DNA extraction was done according to the tissue buffer boiling method. First, 20 µl of tissue buffer (0.25% SDS + 0.05 M NaoH) was mixed with a single colony of a bacterial isolate and the mixture was incubated for 10 minutes in 95°C. After incubation the mixture was centrifuged for 1 minute in 13,000 g and finally 180 µl of Milli-Q water was added and the extracted DNA was frozen in –20°C for long time storage [19], [20].

### Detection of virulence genes encoding type III secretion systems

The virulence genes *exoY*, *exoS*, *exoT*, *exoU* were amplified by the PCR (polymerase chain reaction) method while using specific primers shown in Table 1 (Tab. 1) . Each PCR reaction was done in a total volume of 20 µl as follows: 2 µl of template DNA, 0.6 µl MgCl_2_, 0.4 µl of each primer, 0.2 µl dNTP, 2 µl of 10 × PCR buffer, 0.5 µl of Taq DNA polymerase (5 U/µl) (Fermentase) and 13.9 µl of molecular grade water. The PCR condition was carried out as follows: initial denaturation step (at 94°C for 10 min), followed by 30 to 40 cycles repetitions of denaturation (40 s at 94°C ), annealing (50 s a 57 to 68°C), and extension (55 s at 72°C) with a final extension at 72°C for 10 min [21], [22], [23]. PCR products were analyzed by electrophoresis in 1% of agarose gels for 70–80 min at 100 V. Finally the PCR products were stained with ethidium bromide (0.5 mg/ml) and analyzed by a UV transilluminator.

### Biofilm formation

Quantitative determination of biofilm forming capacity was determined by a colorimetric microtiter plate assay [24]. Briefly, bacterial colonies were grown overnight at 37°C in Trypticase Soy Broth (TSB) (Merck Darmstadt, Germany) for 24 h. The bacterial suspensions were diluted (1:100) in a new TSB medium and 150 µl of this dilution was used to inoculate the sterile flat-bottomed 96-well polystyrene microtiter plates. Subsequent to an incubation period of 24 h at 37°C without shaking, the wells were gently washed three times with 200 µl of PBS (phosphate buffered saline). For the fixation of the biofilms, 100 µl of 99% methanol was added and, after 15 min, the solutions were removed and the plate was air-dried. In the next step, 150 µl of crystal violet 1% (CV) was added to all wells for 20 min. After removing the dye, the bound CV was released with adding 150 µl of 33% acetic acid. The optical density (OD) of each well was measured at 590 nm using a microtiter plate reader. All the assays were repeated for three times. As a control, unioculated medium was used to determine background OD. The cut-off OD (ODc) was defined as three standard deviations above the mean OD of the negative control [25]. All isolates were classified into three groups on the base of OD (ODc) value: OD ≤ ODc = non biofilm producer (-), ODc < OD ≤ 2 * ODc = weak biofilm producer (+), 2* ODc < OD ≤ 4 * ODc = moderate biofilm producer (++), 4* ODc < OD = strong biofilm producer (+++) [26]. All the tests were done triplicate [27].

### Statistical methods

The prevalence of the virulence gene, with respect to the site of infection, was compared by the chi-square test. The correlation between the prevalence of the virulence gene and the antibiotic resistance patterns were tested by the t-test.

## Results

The resistance pattern to the 11 antimicrobials tested is shown in Table 2 (Tab. 2). According to the results, isolates had the lowest rate resistant to polymyxin B and colistin. 

Biofilm data showed that 87% of isolates were biofilm producers in which 69% of them were strongly biofilm producers and the rate of moderate and weakly biofilm producers were 11% and 7%, respectively. 

The type III secretion-toxin encoding gene patterns are shown in Table 3 (Tab. 3). 55% of samples carried *exoY*, 52% of samples carried *exoU*, and 26.3% and 5% carried *exoS* and *exoT*, respectively. 12% of the isolates carried both *exoY* and *exoU* while 32% showed a concomitant existence of *exoS* and *exoY* and 4% carried both *exoS* and *exoU* genes. Coexistence of *exoS*, *exoY*, and *exoU* was seen in 4% of the isolates.

## Discussion

*Pseudomonas aeruginosa* is a common nosocomial pathogen, notorious for its multidrug resistance (MDR) and life threatening infections in critically ill patients. Lately, carbapenems are being used as the last resort antimicrobial treatment for serious infections due to MDR *P. aeruginosa* [28]. In the current study 2.5% of the isolates were resistant to colistin and polymyxin B, which shows that these 2 antibiotics could be in first line drug therapy regimen and the last choice of therapy for these infections. Emergence of resistance to these two antibiotics can treat therapy strategies and there will be no other choice of therapy [29]. 

*P. aeruginosa* secretes four known effector proteins via the type III secretion system: *exoS*, *exoT*, *exoU*, and *exoY* [30]. These proteins modulate host cell functions which are important in cytoskeletal organization and signal transduction [31]. *ExoS* and *exoT* are bifunctional toxins exhibiting ADP-ribosyltransferase and GTPase-activating activity [32]. *ExoT* shows a lower ADP-ribosyltransferase activity than *exoS* [32]. *ExoY* has adenylate cyclase activity whilst *exoU* exhibits phospholipase activity and disrupts eukaryotic membranes following its delivery into the cytoplasm. It has been shown that *exoS* and *exoU* were the major cytotoxins in both in *vitro* and in *vivo* assays [33]. The majority of *P. aeruginosa* strains carry *exoT* and *exoY* genes; however, the presence of *exoS* and *exoU* differ noticeably between the isolates and appear to be mutually exclusive [31]. Different frequencies of cytotoxin encoding genes, however, have been reported in different studies [34]. This may reflects the fact that the genes, encoding the cytotoxins *exoS* and *exoU*, are present as variable traits in *P. aeruginosa* and their presence depends on the disease site or background [35]. Unlike other studies, that show high prevalence of *exoS* and *exoT*, in this study we observe lower prevalence of *exoS* and *exoT *(26.3% and 5%, respectively) (P<0.05) [36]. *ExoY* had the most prevalence (55%) but is found less than in other studies done in Bulgaria (85.8%) and the USA (89%) [21], [37]. In a similar study done in Iran the rate of *exoU* and *exoS* was lower. Jabalameli et al. [38] report a rate of *exoU* as 64.5%, Fazeli et al. [22] in a study on isolates from Iranian hospital infections, report the rate of *exoS* as 67.64% and Dadmanesh et al. [39] publish a *exoS* and *exoT* rate as 73.91% and 69.21% respectively. This lower rate of *exoS* and *exoU* prevalence in our study can be due to less clonal diversity of isolates. Further studies on epidemiological issues can help us understand the pathogenesis of the isolates better. No significant association between MDR resistance and prevalence of the virulence gene carriage was observed (P=0.490).

Biofilm production has been considered to be an important determinant of pathogenicity in *P. aeruginosa* infections [32]. The formation of biofilms facilitate chronic bacterial infections and reduces the efficacy of antimicrobial therapy [23], [32], [40]. In the current study 87% of isolates were biofilm producers and among them 69% of isolates were strong biofilm producers.

50% of the isolates that encode *exoY* (most prevalent in the current study) were the biofilm producer, but only 2.5% of isolates that encode both *exoS* and *exoU* (the major cytotoxins in both in vitro and in vivo assays) were biofilm producers. Interestingly, all non-biofilm producer isolates had at least one of the *exoS* or *exoU* genes. These results can indicate the importance of *exoS* and *exoU* in non-biofilm producer isolates. Also, the *exoY* gene was highly prevalent in biofilm producer isolates. There was no association between the origin of isolates and presence of exo genes (Table 3 (Tab. 3)).

The antibiotic resistant profile of isolates showed increasing resistance, especially in wound and CSF (cerebrospinal fluid) isolates. *P. aeruginosa* isolates from CSF were resistant to all antibiotics, except for colistin. This indicates the importance of antibiotic stewardship development and control of infection in hospital settings. 

In conclusion, findings of the present study showed different distribution of *exo* genes in clinical isolates of *P. aeruginosa* in Northwest Iran. When comparing the presence of *exo* genes and biofilm formation, it was found that *exoS* and *exoU* were more prevalent in non-biofilm producers and *exoY* was more prevalent in biofilm producer isolates. These results indicate the importance of *exoY* in biofilm production of *Pseudomonas aeruginosa*.

## Notes

### Acknowledgment

The present study was funded by the Drug Applied Research Center of the Tabriz University of Medical Sciences. We thank the entire staff of Imam Reza Hospital for their valuable collaboration in sample collection. We also thank the staff of the Department of Medical Microbiology and Virology for their collaboration during the study procedure. This study was done as an Msc thesis of Ms. Somayeh Azimi.

### Competing interests

The authors declare that they have no competing interests.

## Figures and Tables

**Table 1:**
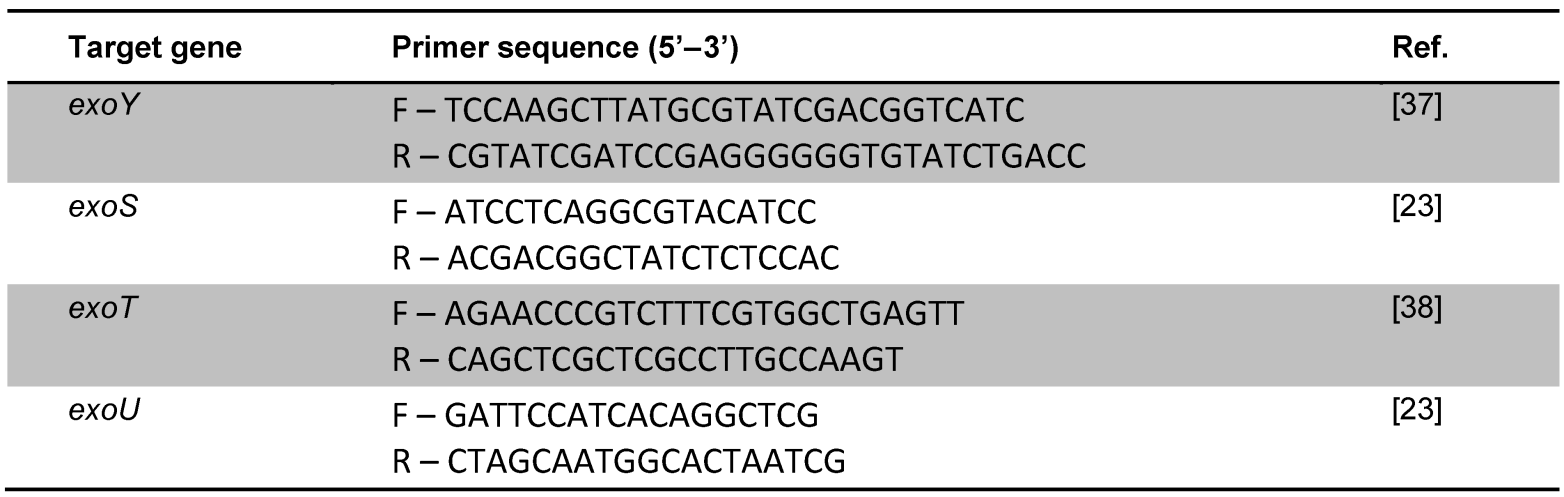
Primers used in the present study for evaluating presence of *exo* genes in *P. aeruginosa* isolates

**Table 2:**
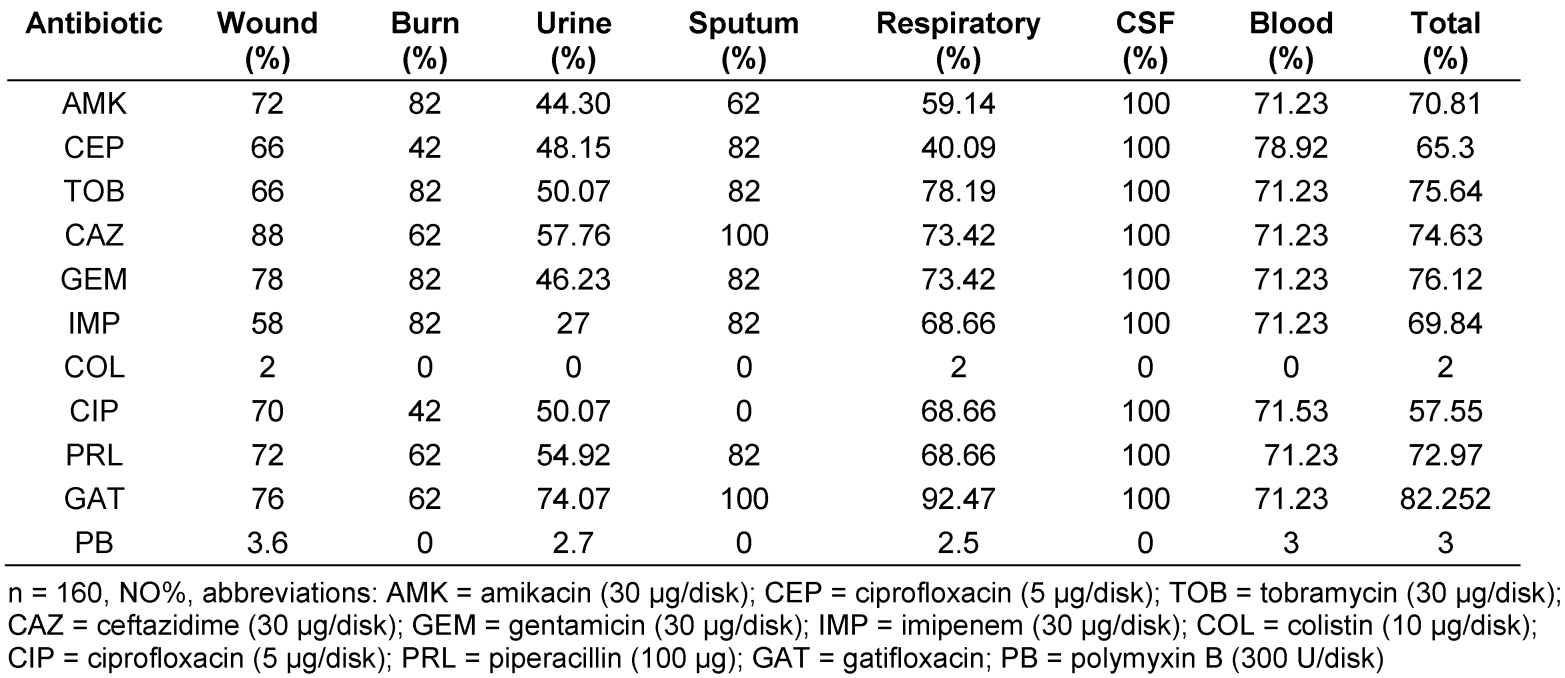
Antimicrobial resistance properties in *Pseudomonas aeruginosa* isolated from clinical infections

**Table 3:**
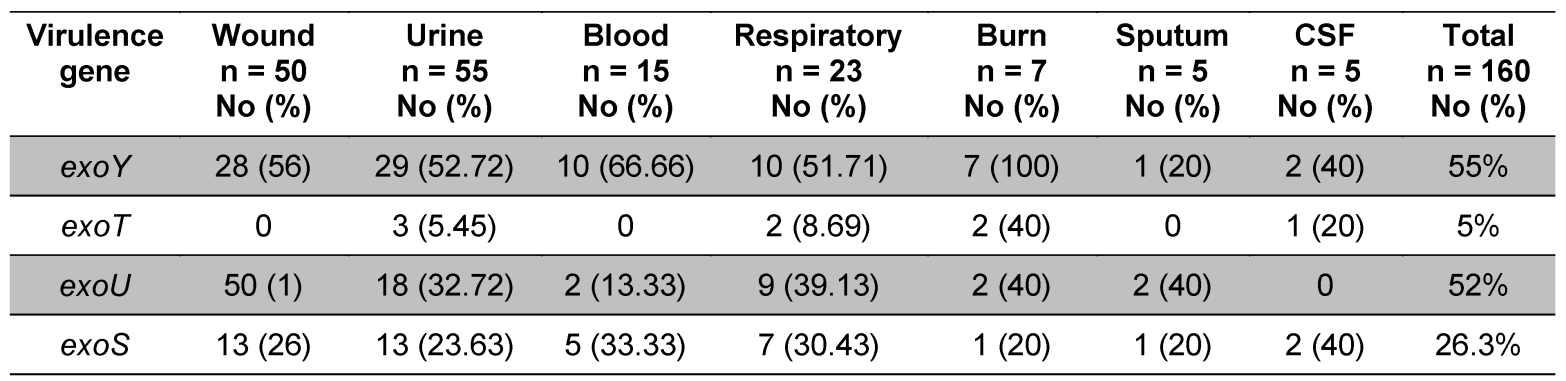
Different distribution of *exo* genes in isolates with different origins
